# Retina tissue validation of optical coherence tomography determined outer nuclear layer loss in FTLD-tau

**DOI:** 10.1186/s40478-021-01290-8

**Published:** 2021-11-18

**Authors:** Benjamin J. Kim, Vivian Lee, Edward B. Lee, Adrienne Saludades, John Q. Trojanowski, Joshua L. Dunaief, Murray Grossman, David J. Irwin

**Affiliations:** 1 Scheie Eye Institute, Department of Ophthalmology, Perelman School of Medicine, University of Pennsylvania, Philadelphia, USA; 2 Translational Neuropathology Research Laboratory, Department of Pathology and Laboratory Medicine, Perelman School of Medicine, University of Pennsylvania, Philadelphia, USA; 3 Center for Neurodegenerative Disease Research, Department of Pathology and Laboratory Medicine, Perelman School of Medicine, University of Pennsylvania, Philadelphia, USA; 4 Frontotemporal Lobar Degeneration Center, Department of Neurology, Perelman School of Medicine, University of Pennsylvania, Philadelphia, USA

## Abstract

Alzheimer’s disease (AD) is associated with inner retina (nerve fiber and ganglion cell layers) thinning. In contrast, we have seen outer retina thinning driven by photoreceptor outer nuclear layer (ONL) thinning with antemortem optical coherence tomography (OCT) among patients considered to have a frontotemporal degeneration tauopathy (FTLD-Tau). Our objective was to determine if postmortem retinal tissue from FTLD-Tau patients demonstrates ONL loss observed antemortem on OCT. Two probable FTLD-Tau patients that were deeply phenotyped by clinical and genetic testing were imaged with OCT and followed to autopsy. Postmortem brain and retinal tissue were evaluated by a neuropathologist and ocular pathologist, respectively, masked to diagnosis. OCT findings were correlated with retinal histology. The two patients had autopsy-confirmed FTLD-Tau neuropathology and had antemortem OCT measurements showing ONL thinning (66.9 μm, patient #1; 74.9 μm, patient #2) below the 95% confidence interval of normal limits (75.1–120.7 μm) in our healthy control cohort. Postmortem, retinal tissue from both patients demonstrated loss of nuclei in the ONL, matching ONL loss visualized on antemortem OCT. Nuclei counts from each area of ONL loss (2 – 3 nuclei per column) seen in patient eyes were below the 95% confidence interval (4 – 8 nuclei per column for ONL) of 3 normal control retinas analyzed at the same location. Our evaluation of retinal tissue from FTLD-Tau patients confirms ONL loss seen antemortem by OCT. Continued investigation of ONL thinning as a biomarker that may distinguish FTLD-Tau from other dementias is warranted.

## Introduction

Tauopathies are a class of frontotemporal lobar degeneration proteinopathies (FTLD-Tau) commonly associated with frontotemporal dementia (FTD) syndromes. Clinical distinction of FTLD-Tau from other FTLD-associated proteinopathies (i.e. FTLD-TDP, FTLD-FUS) and Alzheimer’s disease (AD) is challenging [8]. Thus, non-invasive biomarkers indicative of histologic features of FTLD-Tau are urgently needed to improve diagnosis and facilitate therapeutic trials.

Optical coherence tomography (OCT) enables visualization of neuronal tissue in vivo. Many studies have found inner retina thinning (nerve fiber and ganglion cell layer) in AD vs. controls [3]. In contrast, our OCT studies revealed outer retina thinning with normal inner retina thicknesses in living FTD patients with clinical features or genetic mutations predictive of FTLD-Tau pathology [12, 13, 18]. The outer retina thinning is driven by loss of the outer nuclear layer (ONL), which consists of photoreceptor nuclei and composes most of the outer retina’s thickness. This outer retina thinning correlates with global cognitive impairment, and longitudinal OCT analysis found persistent and progressive ONL thinning among the probable FTLD-Tau patients [12, 13].

Postmortem retinal tissue confirmation of antemortem OCT findings is paramount to the potential development of OCT as a biomarker, but this data is lacking in FTD. Here, we report the first 2 consecutive autopsy findings of our OCT imaged FTLD-Tau patients for important postmortem validation.

## Methods

Patients were followed in a clinical research program at the Penn Frontotemporal Degeneration Center (FTDC) and Scheie Eye Institute of the University of Pennsylvania. Clinical diagnosis was established at FTDC using modern clinical criteria for FTD syndromes at weekly consensus meetings as previously described [12, 13]. Patients had genetic testing for pathogenic mutations in *MAPT* (OMIM: 157140), progranulin (*GRN*) (OMIM:138945), *C9orf72* (OMIM: 61426), and other FTD related genes based on pedigree analysis as previously described [21]. Ophthalmic evaluation included a full, dilated eye exam by a retina specialist (BJK). All patients had no history of disease that would affect retinal thickness measurements, including diabetes, retinal or optic nerve disease, high refractive error (± 6.00 diopter spherical equivalent), or intraocular surgery (e.g. cataract surgery) within 90 days of the eye exam. Procedures for the OCT protocol, and neuropathological diagnosis have been previously described [12, 13, 19]. Briefly, patients were imaged with a standard spectral-domain OCT protocol using the Heidelberg Spectralis (Franklin, MA, USA). Each patient had a macular volume scan with 20 degree images, 25 raster scans, and automated real time averaging of 25 scans for each raster scan. While masked to clinical information, an analyst then segmented the retinal layers using the automated Iowa Reference Algorithm (v3.6), and segmentation errors were manually corrected [1, 6, 10]. Retinal layer thickness measurements were reported after averaging the values of the central 5 regions of the ETDRS (Early Treatment of Diabetic Retinopathy Study) grid.

Autopsies were performed at the Center for Neurodegenerative Disease Research with post-mortem intervals < 12 h. At autopsy, eyes and brain were collected. Independent evaluation of eyes and brains was performed by an ophthalmic pathologist (VL) and neuropathologist (EBL), respectively. Eyes were fixed in 10% formalin, processed, and paraffin embedded; five μm sections were stained with hematoxylin and eosin. The ophthalmic pathologist performed histologic retinal evaluation while masked to clinical and neuropathological diagnosis. Representative sections of the whole eye were reviewed. As the study goal was to validate ONL loss seen on OCT, the evaluation of slides was directed towards the macula, where each eye was sectioned serially at multiple levels of the macula and evaluated in a uniform fashion.

Nuclei counts for the inner nuclear layer (INL) and ONL are an accepted way to evaluate retinal tissue [2, 16, 22]. In contrast, ganglion cell layer nuclei counts are known to have wide variability among normals [7], making them difficult to compare within small groups. After identifying areas of suspected macular ONL thinning, 20 – 30 columns of ONL and INL nuclei were counted at intervals of 10 microns. As measurements are affected by retinal location [2, 16, 22], the nuclei counts for each eye were then compared to ONL and INL nuclei counts at macular areas of equivalent size, location, and distance from the optic nerve from 3 different normal controls. Controls were 3 consecutive eyes obtained from the National Disease Research Interchange (Philadelphia, PA) and were from 3 subjects with no history of eye disease that would affect the retina, diabetes, or a neurodegenerative condition.

Suspected nuclei loss, either on OCT or histopathology, was considered abnormal if the measurement was outside the 95% confidence interval (95%CI) of normal limits using the normal approximation method (e.g., mean ± (1.96 X standard deviation)). Normal references were calculated for OCT data using previously reported control data [12], and they were calculated for retinal tissue nuclei counts from the 3 normal control retinas, yielding a normal range consistent with published nuclei counts of normal retinas [14].

This study was approved by the University of Pennsylvania Institutional Review Board and followed the tenets of the Declaration of Helsinki. All patients gave written informed consent (with caregivers if indicated).

## Results

### Clinical evaluation and imaging

Patient #1 was a 63-year-old Caucasian woman diagnosed with progressive supranuclear palsy (PSP) after 3 years of progressive L-DOPA resistant Parkinsonism, falls, and executive limitations with later emerging expressive language deficits. OCT was performed prior to this patient enrolling in a trial (NCT03068468) for PSP; she received gosuranemab (Biogen), a humanized antibody that binds N-terminal tau. Her symptoms progressed to end-stage dementia and death at age 67. Patient #2 was a 58-year-old Caucasian woman diagnosed with the behavioral variant of FTD (bvFTD) after 5 years of progressive cognitive and behavioral symptoms, as well as features of single-word and object knowledge later in her disease course from severe anterior temporal lobe disease. She had a pathogenic mutation in *MAPT (*E10 + 16 C > T mutation) and declined clinically with features of Parkinsonism and mutism with death at age 64 from end-stage dementia.

**Fig. 1:**
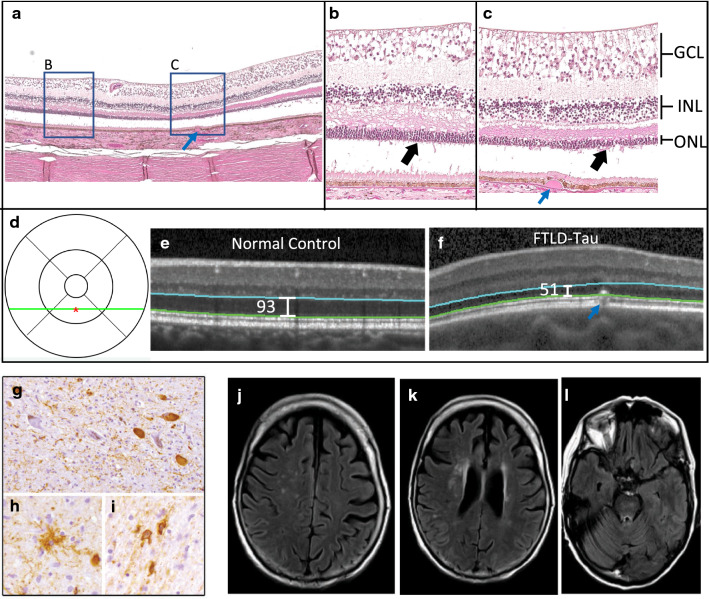
Retinal tissue of patient #1 showing outer nuclear layer (ONL) loss with spatial correlation to ONL thinning seen on optical coherence tomography (OCT) and associated brain pathology. Left eye retinal tissue from inferior parafoveal macula (**a**) (H & E, original magnification X 4). This patient had a normal macula with a single druse (blue arrow) at the macula which enabled precise localization of retinal tissue. Boxes show corresponding areas of higher magnification (**b**, **c**) (original magnification X 20). This patient has some normal appearing ONL (area **b**, black arrow) (about 6 layers of nuclei), but area **c** shows an area of ONL loss (black arrow) (2–4 layers of nuclei). Other retinal layers have no abnormalities, except for typical tissue processing artifactual separation of photoreceptor segments from the ONL and partial separation of INL from the outer plexiform layer. There is no artifact affecting ONL nuclei. Full retina examination prior to expiration showed a normal appearing macula with no confounding retinal disease. OCT at that time also showed macula ONL thinning, especially at inferior macula. **d** shows the ETDRS grid location of inferior parafoveal OCT scans (**e**, ** f**) (green line) and of point measurements (red asterisk). **e** shows OCT of an age, sex, race matched normal control with normal ONL thickness in comparison to ONL thinning seen on this patient’s OCT in which the same druse (blue arrow) seen in retinal tissue is visualized (**f**). OCT image ONL boundaries are shown in blue and green; Heidelberg Spectralis (Franklin, MA) caliper measurements are shown. Brain pathology showed four-repeat (4R) tauopathy with threads (**g**), tufted astrocytes (**h**), tangles (**g**) and white matter coiled bodies within oligodendrocytes (**i**) consistent with progressive supranuclear palsy. MRI (**j**, **k**, **l**) showed prominent frontal lobe atrophy that was asymmetric and most prominent in left medial and dorsolateral frontal lobes. *GCL* ganglion cell layer, *INL* inner nuclear layer, *ONL* outer nuclear layer

**Fig. 2:**
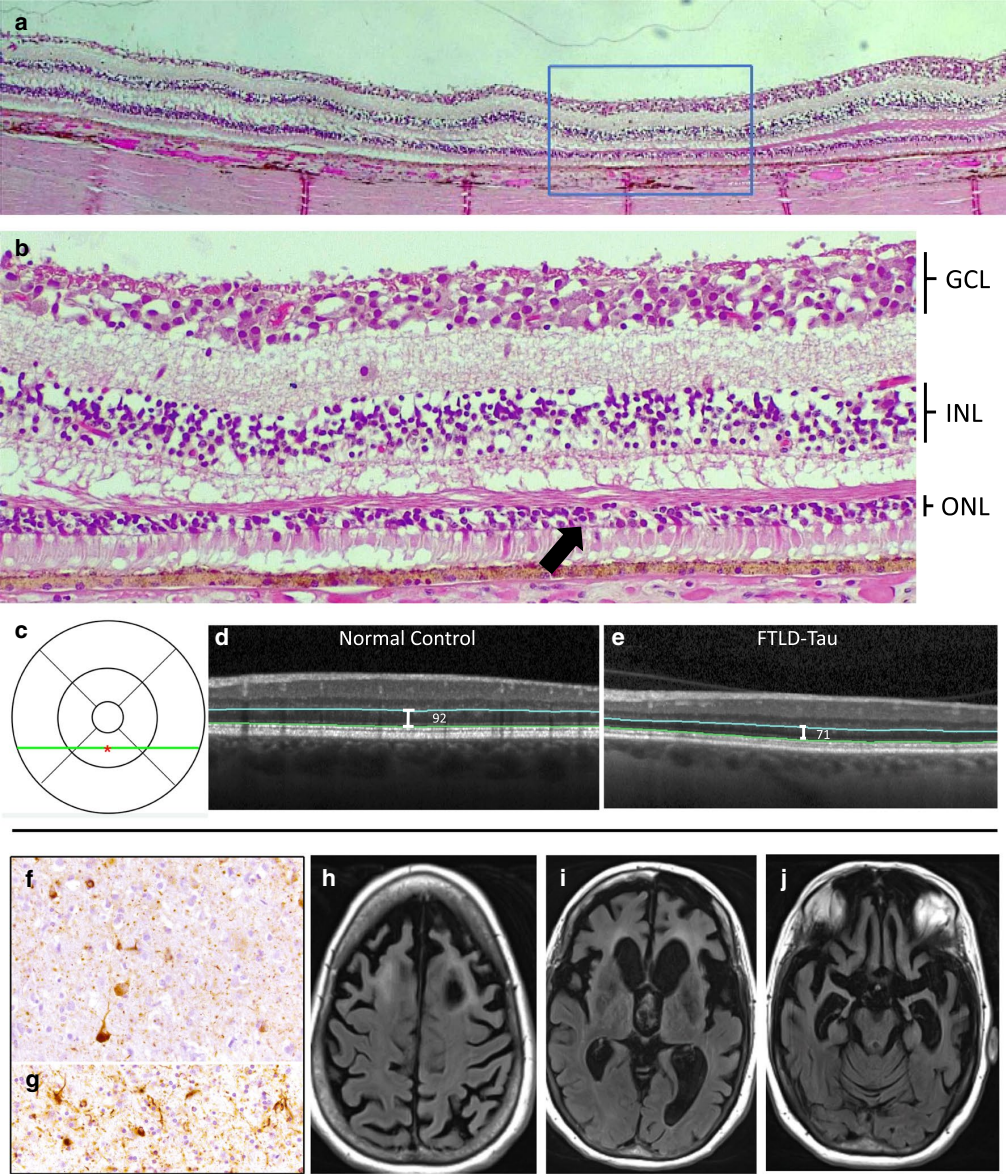
Retinal tissue of patient #2 showing outer nuclear layer (ONL) loss, correlation to ONL thinning seen on optical coherence tomography, and associated brain pathology. Left eye retina tissue from macula **a** (H & E, original magnification X 5). Box shows corresponding area of higher magnification **b** (original magnification X 20). There is a significant area of ONL thinning (black arrow) with only 2–4 layers of nuclei. Other retinal layers have no abnormalities except for mild tissue processing artifact. There is no artifact affecting ONL nuclei. Full retina examination prior to expiration showed a normal appearing macula with no confounding retinal disease. OCT at that time also showed macula ONL thinning. **c** shows the ETDRS grid location of inferior parafoveal OCT scans **d**, **e** (green line) and of point measurements (red asterisk). **d** shows OCT of an age, sex, race matched normal control with normal ONL thickness in comparison to ONL thinning seen on this patient’s OCT (**e**). Brain pathology displayed tau pathology consisting of pretangles (**f**), tangles (**f**), grains (**f**, **g**) and white matter coiled bodies (**g**) consistent with frontotemporal dementia with parkinsonism linked to chromosome 17. MRI (**h**, **i**, **j**) showed severe widespread frontotemporal atrophy bilaterally. *GCL* ganglion cell layer, *INL* inner nuclear layer, *ONL* outer nuclear layer

Each patient’s antemortem ophthalmic exam was normal and OCT images showed no known eye disease. With our imaging protocol, normal thicknesses for the retinal nerve fiber layer (RNFL), ganglion cell layer (GCL), and outer nuclear layer (ONL) thickness are 23 μm, 41 μm, and 98 μm, respectively [12]. As shown in Table 1, these patients had normal inner retina (RNFL and GCL) thicknesses. However, both patients had ONL thinning which was outside the 95% confidence interval thickness in our normal controls [12] (66.9 μm for left eye of patient 1 and 74.9 μm for right eye of patient 2), while the contralateral eye had thinning approaching statistical significance, especially for patient #2. With MRI, patient #1 demonstrated asymmetric frontal lobe atrophy (Fig. 1), and patient #2 had more widespread frontotemporal atrophy bilaterally (Fig. 2).

**Table 1:** Clinical data and neuropathologic diagnosis

Patient	Primary Clinical Diagnosis	Age at Time of Eye Exam, Sex, Race	Visual acuity	Case RNFL	Normal RNFL^a^ (95%CI)	Case GCL	Normal GCL^a^ (95%CI)	Case ONL	Normal ONL^a^ (95%CI)	Age at expiration	Primary neuropathology diagnosis	PMI (hrs)	Brain Wt. (g)
1	PSP	63 Female Caucasian	20/25 OD 20/30 OS	21.4 OD, 20.1 OS	23.2 (15.1–31.3)	36.1 OD, 40.1 OS	40.7 (17.9–63.5)	89.6 OD, **66.9** OS	97.9 (75.1–120.7)	67	PSP^b^	7	1177
2	bvFTD	58 Female Caucasian	20/25 OU	21.8 OD, 22.9 OS		28.9 OD, 34.2 OS		**74.9** OD, 79.9 OS		64	FTDP-17 *MAPT* mutation c.915 + 16C > T^c^	7.5	1027

*RNFL* retinal nerve fiber layer, *GCL* ganglion cell layer, *ONL* outer nuclear layer, *PSP* progressive supranuclear palsy, *bvFTD* behavior-variant frontotemporal dementia, *PMI* postmortem interval in hours, *Brain Wt* brain weight in grams

^a^Based on Kim BJ et al., *Neurology* 2017 [12]

^b^Additional low burden of diffuse plaque (Thal stage A1)

^c^Additional low burden of diffuse plaque (Thal stage A2)

### Autopsy data

Postmortem brain examination of patient #1 demonstrated tau-positive threads, tufted astrocytes, coiled bodies, and perivascular vesicular astrocytes consistent with PSP with immunotherapy treatment effect (Fig. 1) [11]. Patient #2 exhibited tau-positive pretangles, tangles, grains, and coiled bodies consistent with her known *MAPT* mutation (Fig. 2).

Evaluation of the retinas revealed no inner retina abnormalities. Histologically, outer retina health is commonly measured by ONL nuclei thickness. Normal maculas in the 6th–7th decade of life typically have about 6 layers of ONL nuclei [14]. For patient #1, the right eye did not have appreciable ONL loss, but the left eye had definite ONL loss (range of 2–4 layers of nuclei) at the inferior macula, where the most ONL thinning was seen by OCT (Fig. 1). Both eyes of Patient #2 had ONL loss (range of 2–4 layers of nuclei) within the macula corresponding to OCT findings (Fig. 2 shows left eye).

When each of these areas of ONL loss were compared to equivalent macular locations in 3 normal controls (Table 2), the ONL nuclei counts were below the 95% confidence interval of controls (Table 3), but the INL nuclei counts of these areas were within the normal range.

**Table 2:** Demographic data of normal control retinal tissue donors

Control	Age (years)	Sex	Race	PMI (hrs)	Cause of Death
1	70	Male	Caucasian	16.5	Cardiac Arrest
2	84	Female	Caucasian	4.75	Respiratory Failure
3	85	Male	Caucasian	23.5	Cardiac Arrest

**Table 3:** Outer nuclear layer and inner nuclear layer nuclei counts for patient eyes and controls

FTLD-Tau patient	Eye	ONL nuclei count of patient eye	Average ONL nuclei count of 3 control eyes (95% CI)	INL nuclei count of patient eye	Average INL nuclei count of 3 control eyes (95% CI)
1	OD^a^	6.0	6.0^a^ (3.8–8.1)	3.5	5.0^a^ (2.4–7.5)
	OS^a^	3.1	6.0^a^ (3.8–8.1)	6.1	5.0^a^ (2.4–7.5)
2	OD^a^	3.2	6.0^a^ (3.8–8.1)	7	5.0^a^ (2.4–7.5)
	OS^b^	2.4	5.9^b^ (3.6–8.3)	5.2	4.1^b^ (2.3–5.9)

*ONL* outer nuclear layer, *INL* inner nuclear layer, *CI* confidence interval

^a^Retinal location: 2.0 mm from optic nerve, inferior macula

^b^Retinal Location: 4.6 mm from optic nerve, inferior macula

## Discussion

Although OCT is considered highly reproducible, there is some variability or conflicting reports on OCT findings in neurodegenerative diseases. This is contributed to, in part, by the need for gold-standard autopsy confirmation to validate the patient’s specific underlying disease. This is especially true for the heterogeneous spectrum of FTLD. Therefore, tissue confirmation of OCT findings is critically important. We have presented novel and rare postmortem histopathological analysis of retinal tissue digitally quantified using OCT during life and correlated with underlying FTLD neuropathologic diagnosis. Our tissue data corroborates an outer retina abnormality seen on OCT among dementia patients. In comparison, other studies have shown retinal tissue with inner retina loss for AD [7] and normal OCT outer retina thickness in AD patients vs. controls [20].

While these patients presented with different clinical syndromes associated with tauopathies, our autopsy data aligns with our published OCT data suggesting a link between ONL thinning and FTLD-Tau, independent of clinical diagnosis [12, 13]. For each of the FTLD-Tau patients, significant ONL thinning was seen but there was some asymmetry with milder trends in the contralateral eye, especially for patient #1 who also had asymmetry of cortical atrophy on MRI. Indeed, FTLD microscopic pathology and antemortem atrophy often is distributed asymmetrically in the cortex [9, 17] and may influence the mild laterality of OCT data in our sample here. Nonetheless, we observed significant ONL loss postmortem that corresponds to in vivo OCT data. Our observations of areas of ONL loss were supported further by an analysis of nuclei counts showing abnormally low nuclei counts within the ONL, but normal nuclei counts within the nearby INL compared to controls. The mechanisms for tau-mediated neurodegeneration affecting the ONL are unclear, but tau has been shown within human photoreceptors [15]. While the cause of ONL thinning is unknown, it may reflect phosphorylated tau toxicity related to the unusual amount of oxidative stress photoreceptors encounter [4]. Among FTLD-Tau patients, oxidative stress may promote the phosphorylation of tau within photoreceptors, which in turn may have a toxic effect on photoreceptors and explain preferential damage to photoreceptors as opposed to other neuronal cells of the retina.

The small number of cases is the primary limitation of this data and deserves emphasis. Nevertheless, we believe our rare brain and eye autopsy data from these consecutive cases is compelling as it is entirely consistent with our prior studies suggesting ONL thinning in FTLD-Tau. There was limited availability of normal control retinal tissue, and the eyes were from subjects of greater age than the FTLD-Tau patients. However, these subjects had nuclei counts within the expected normal range, and age is associated with mild ONL loss (not an increase) [5], providing a fair comparison to our patients to support the finding of significant ONL loss. We also acknowledge that patient #1 received anti-tau immunotherapy with unclear effects on underlying biology and patient #2 had hereditary tauopathy. Thus, additional autopsy samples are needed to confirm findings, test generalization for sporadic tauopathies, and contrast with clinically similar AD and FTLD-TDP.

To our knowledge, these data are the first human tissue confirmation of OCT abnormalities in FTD, and the first tissue confirmation of an outer retina abnormality seen in dementia patients examined and imaged with OCT during life. These data support the continued investigation of retina imaging as a biomarker that may distinguish FTLD-Tau patients from other dementias including Alzheimer’s disease.

## Data Availability

The datasets used and/or analysed during the current study are available from the corresponding author on reasonable request.

## Abbreviations

AD: Alzheimer’s disease
FTD: Frontotemporal degeneration
FTLD-Tau: Frontotemporal lobar degeneration tauopathy
FTLD-TDP: Frontotemporal lobar degeneration TAR DNA binding protein 43
GCL: Ganglion cell layer
INL: Inner nuclear layer
OCT: Optical coherence tomography
ONL: Outer nuclear layer
PSP: Progressive supranuclear palsy
RNFL: Retinal nerve fiber layer

## References

[1] Abramoff MD, Garvin MK, Sonka M (2010). Retinal imaging and image analysis. IEEE Rev Biomed Eng.

[2] Baumann BH, Shu W, Song Y, Sterling J, Kozmik Z, Lakhal-Littleton S (2019). Liver-specific, but not retina-specific, hepcidin knockout causes retinal iron accumulation and degeneration. Am J Pathol.

[3] Chan VTT, Sun Z, Tang S, Chen LJ, Wong A, Tham CC (2019). Spectral domain-optical coherence tomography measurements in Alzheimer’s disease: a systematic review and meta-analysis. Ophthalmology.

[4] Fridlich R, Delalande F, Jaillard C, Lu J, Poidevin L, Cronin T (2009). The thioredoxin-like protein rod-derived cone viability factor (RdCVFL) interacts with TAU and inhibits its phosphorylation in the retina. Mol Cell Proteom.

[5] Gartner S, Henkind P (1981). Aging and degeneration of the human macula. 1. Outer nuclear layer and photoreceptors. Br J Ophthalmol.

[6] Garvin MK, Abramoff MD, Wu X, Russell SR, Burns TL, Sonka M (2009). Automated 3-D intraretinal layer segmentation of macular spectral-domain optical coherence tomography images. IEEE Trans Med Imaging.

[7] Hinton DR, Sadun AA, Blanks JC, Miller CA (1986). Optic-nerve degeneration in Alzheimer’s disease. N Engl J Med.

[8] Irwin DJ, Cairns NJ, Grossman M, McMillan CT, Lee EB, Van Deerlin VM (2015). Frontotemporal lobar degeneration: defining phenotypic diversity through personalized medicine. Acta Neuropathol.

[9] Irwin DJ, McMillan CT, Xie SX, Rascovsky K, Van Deerlin VM, Coslett HB (2018). Asymmetry of post-mortem neuropathology in behavioural-variant frontotemporal dementia. Brain.

[10] Kang L, Wu X, Chen DZ, Sonka M (2006). Optimal surface segmentation in volumetric images: a graph-theoretic approach. IEEE Trans Pattern Anal Mach Intell.

[11] Kim B, Mikytuck B, Suh E, Gibbons GS, Van Deerlin VM, Vaishnavi SN (2021). Tau immunotherapy is associated with glial responses in FTLD-Tau. Acta Neuropathol.

[12] Kim BJ, Irwin DJ, Song D, Daniel E, Leveque JD, Raquib AR (2017). Optical coherence tomography identifies outer retina thinning in frontotemporal degeneration. Neurology.

[13] Kim BJ, Grossman M, Song D, Saludades S, Pan W, Dominguez-Perez S (2019). Persistent and progressive outer retina thinning in frontotemporal degeneration. Front Neurosci.

[14] Kim SY, Sadda S, Pearlman J, Humayun MS, de Juan E, Melia BM (2002). Morphometric analysis of the macula in eyes with disciform age-related macular degeneration. Retina.

[15] Leger F, Fernagut P, Canron M, Leoni S, Vital C, Tison F (2011). Protein aggregation in the aging retina. J Neuropathol Exp Neurol.

[16] Masri RA, Weltzien F, Purushothuman S, Lee SCS, Martin PR, Grunert U (2021). Composition of the inner nuclear layer in human retina. Invest Oph Vis Sci.

[17] Mesulam MM, Weintraub S, Rogalski EJ, Wieneke C, Geula C, Bigio EH (2014). Asymmetry and heterogeneity of Alzheimer's and frontotemporal pathology in primary progressive aphasia. Brain.

[18] Sun JQ, McGeehan B, Firn K, Irwin D, Grossman M, Ying GS (2020). Comparison of the Iowa Reference Algorithm to the Heidelberg Spectralis optical coherence tomography segmentation algorithm. J Biophotonics.

[19] Toledo JB, Van Deerlin VM, Lee EB, Suh ER, Baek Y, Robinson JL (2014). A platform for discovery: The University of Pennsylvania Integrated Neurodegenerative Disease Biobank. Alzheimers Dement.

[20] Uchida A, Pillai JA, Bermel R, Bonner-Jackson A, Rae-Grant A, Fernandez H (2018). Outer retinal assessment using spectral-domain optical coherence tomography in patients with alzheimer’s and parkinson’s disease. Invest Ophthalmol Vis Sci.

[21] Wood EM, Falcone D, Suh E, Irwin DJ, Chen-Plotkin AS, Lee EB (2013). Development and validation of pedigree classification criteria for frontotemporal lobar degeneration. JAMA Neurol.

[22] Zhao L, Wang C, Song D, Li Y, Song Y, Su G (2014). Systemic Administration of the Antioxidant/Iron Chelator alpha-Lipoic Acid Protects Against Light-Induced Photoreceptor Degeneration in the Mouse Retina. Invest Oph Vis Sci.

